# Characterization of photosynthetic gas exchange in leaves under simulated adaxial and abaxial surfaces alternant irradiation

**DOI:** 10.1038/srep26963

**Published:** 2016-07-05

**Authors:** Zi-Shan Zhang, Yu-Ting Li, Hui-Yuan Gao, Cheng Yang, Qing-Wei Meng

**Affiliations:** 1 State Key Lab of Crop Biology, Tai’an, Shandong Province, China; 2 College of Horticulture Science and Engineering, Shandong Agricultural University, Tai’an, Shandong Province, China; 3 College of Life Sciences, Shandong Agricultural University, Tai’an, Shandong Province, China; 4 Wheat Research Institute, Henan Academy of Agricultural Sciences, Zhengzhou, Henan 450002, China

## Abstract

Previous investigations on photosynthesis have been performed on leaves irradiated from the adaxial surface. However, leaves usually sway because of wind. This action results in the alternating exposure of both the adaxial and abaxial surfaces to bright sunlight. To simulate adaxial and abaxial surfaces alternant irradiation (ad-ab-alt irradiation), the adaxial or abaxial surface of leaves were exposed to light regimes that fluctuated between 100 and 1,000 μmol m^−2^ s^−1^. Compared with constant adaxial irradiation, simulated ad-ab-alt irradiation suppressed net photosynthetic rate (Pn) and transpiration (E) but not water use efficiency. These suppressions were aggravated by an increase in alternant frequency of the light intensity. When leaves were transferred from constant light to simulated ad-ab-alt irradiation, the maximum Pn and E during the high light period decreased, but the rate of photosynthetic induction during this period remained constant. The sensitivity of photosynthetic gas exchange to simulated ad-ab-alt irradiation was lower on abaxial surface than adaxial surface. Under simulated ad-ab-alt irradiation, higher Pn and E were measured on abaxial surface compared with adaxial surface. Therefore, bifacial leaves can fix more carbon than leaves with two “sun-leaf-like” surfaces under ad-ab-alt irradiation. Photosynthetic research should be conducted under dynamic conditions that better mimic nature.

Bifacial leaves are heterogeneous. They have two distinct surfaces: the adaxial surface and the abaxial surface. It was widely believed that the adaxial surface is the main contributor to carbon gain because of its higher photosynthetic capacity and the preferential irradiation of the adaxial surface^1^,^2^. The adaxial surface intercepts light more efficiently than the abaxial surface because the palisade mesophyll (PM) cells of the adaxial surface are packed more closely than the spongy mesophyll (SM) cells near the abaxial surface^3^. The highest photosynthetic rates are observed in the middle and lower palisade layers^4^,^5^,^6^, which have higher electron transport activity and more photosynthetic proteins^7^,^8^. The adaxial surface also has a stronger photoprotection mechanism and an increased resistance to photoinhibition^9^. The adaxial surface is considered to have sun-leaf-like characteristics and the abaxial surface is considered to have shade-leaf-like characteristics^10^.

In addition, it is often empirically believed that the adaxial surface is exposed to more direct radiation, whereas the abaxial surface is shaded by the leaf itself and receives weaker light that is transmitted through the mesophyll and reflected from the surroundings^1^. However, leaves usually sway because of external forces such as wind. As a result, the adaxial and abaxial surfaces are alternately exposed to bright sunlight (Fig. 1). This phenomenon is more prevalent in tall trees and plants in windy areas^11^.

Consequently, to understand photosynthesis under natural conditions, we need to understand photosynthesis under adaxial and abaxial surfaces alternant irradiation conditions (ad-ab-alt irradiation). Unfortunately, previous studies examining the photosynthetic performance of leaves were performed using adaxial surface irradiation^12^,^13^,^14^,^15^,^16^,^17^. Less attention has been paid to the photosynthetic performance of leaves under abaxial surface irradiation conditions^9^,^18^, and no attention has been given to investigating the photosynthetic gas exchange of leaves under ad-ab-alt irradiation.

Most of the photosynthetically active radiation (PAR, 400-700 nm; over 90% blue light and 70% red light) is absorbed by the surface facing the incident light. Only green light that has low efficiency in photosynthesis can be received by the other surface^19^,^20^. Therefore, both the adaxial surface and the abaxial surface of swaying leaves experience fluctuating light conditions between high and low light, similar to a sunfleck. It has been proved that the photosynthetic performance of leaves under sunfleck conditions and constant irradiation are different^21^,^22^. The deactivation of Rubisco^23^ and stomatal closure^24^,^25^ under low light will result in the depression of photosynthetic gas exchange during the following high light sunfleck period. We propose that photosynthetic gas exchange, such as CO_2_ fixation and transpiration, should be repressed with ad-ab-alt irradiation. In addition, due to the different protein and pigment distributions between the adaxial and abaxial surfaces^5^,^8^, we expect differential responses to ad-ab-alt irradiation between the adaxial and abaxial surfaces of an individual leaf.

To test these hypotheses, we examined dynamic gas exchange in the leaves of two trees (*Platanus orientalis* L. and *Melia azedarach* L.) and one herb (*Solanum lycopersicum* L.) under simulated ad-ab-alt irradiation or constant irradiation.

## Results

### Photosynthetic gas exchange and absorptance of leaves under constant light

The steady-state photosynthetic gas exchange of the leaves was measured when the adaxial or abaxial surface was exposed to constant high light (1,000 μmol m^−2 ^s^−1^; ad-con irradiation or ab-con irradiation). The steady-state net photosynthetic rate (Pn_S_), transpiration rate (E_S_) and water use efficiency (WUE_S_) of the three species under ad-con irradiation were significantly higher than under ab-con irradiation. In *Solanum lycopersicum* L. and *Platanus orientalis* L. leaves, the E_S_ was similar under ad- and ab-con irradiation (Supplementary Fig. S1). These results support the hypothesis that the adaxial and abaxial surfaces of bifacial leaves demonstrate the respective characteristics of sun and shade leaves^3^,^26^,^27^. The PAR absorptance of the leaves was similar under ad- and ab-con irradiation (Supplementary Fig. S1). This finding suggests that the differences in photosynthetic gas exchange under adaxial or abaxial surface irradiation were independent of the absorptance.

### Gas exchange of leaves under simulated ad-ab-alt irradiation

**Figure 1:**
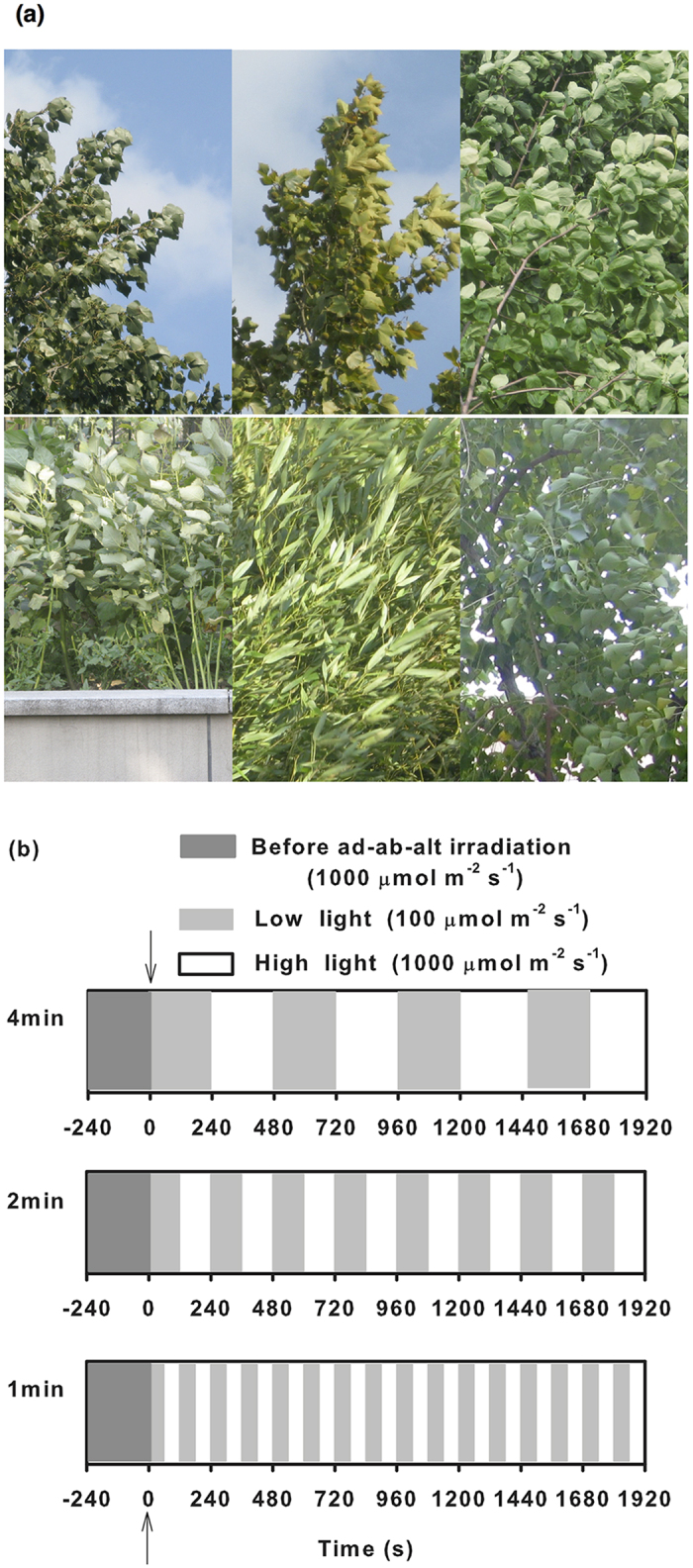
Photographs showing the abaxial surface exposed to the sun (**a**) and a sketch map of the fluctuating light regimes used in this study (**b**). In plot (**a**), the species in the top row are *Populus ussuriensis* Kom., *Platanus orientalis*, and *Magnolia denudata* Desr. (from left to right), and the species in the bottom row are *Agastache rugosa*, *Bambusoideae* species and *Populus cathayana* Rehd. (from left to right). In plot (**b**), the leaves were adapted under high light (1,000 μmol m^−2^ s^−1^) for 20–40 min until Pn and E stabilized (dark grey). The leaves were exposed to ad-ab-alt irradiation at 0 s (marked with an arrowhead). During the simulated ad-ab-alt irradiation, the irradiation was switched between high light (1,000 μmol m^−2^ s^−1^) and low light (100 μmol m^−2^ s^−1^) every 240 s, 120 s or 60 s. The high light periods (white segments) were used to calculate the integrated gas exchange.

**Figure 2:**
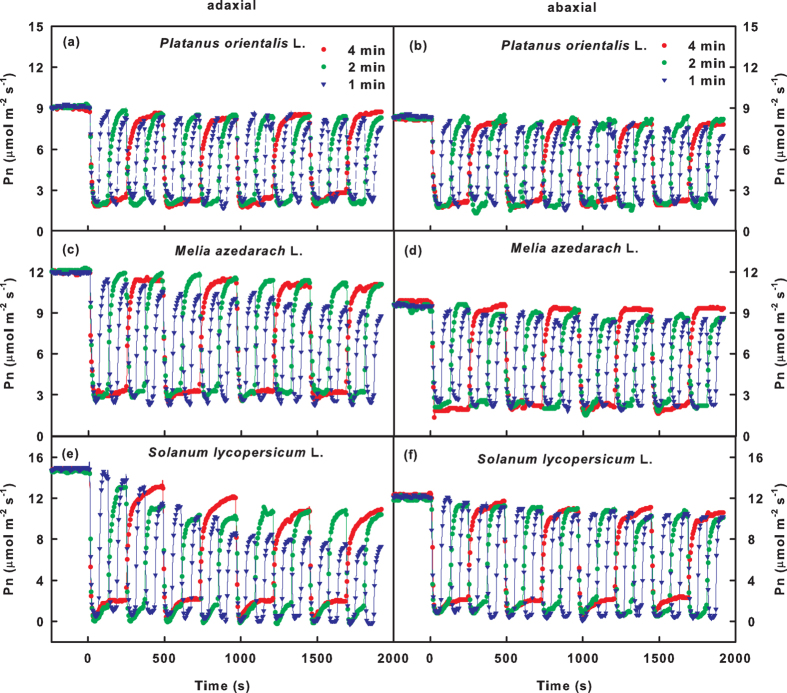
The time course of the net photosynthetic rates (Pn) of leaves of *Platanus orientalis* L. (**a,b**), *Melia azedarach* L. (**c,d**) and *Solanum lycopersicum* L. (**e,f**) when the adaxial (**a,c,e**) or abaxial surface (**b,d,f**) was irradiated under fluctuating light. The irradiation was switched between high light (1,000 μmol m^−2^ s^−1^) and low light (100 μmol m^−2 ^s^−1^) every 240 s, 120 s or 60 s.

To simulate the ad-ab-alt irradiation, the adaxial or abaxial surface was irradiated by fluctuating light (Fig. 1). Under the fluctuating light condition, low light (100 μmol m^−2^ s^−1^) and high light (1,000 μmol m^−2^ s^−1^) were alternated every 60 s, 120 s or 240 s (low, medium or high frequency fluctuating light, respectively; Fig. 1). The representative traces of dynamic gas exchange in the leaves of the three species are shown in Fig. 2 and Supplementary Fig. S2.

When the light intensity was switched from high light to low light, Pn immediately decreased by 80–95% and slightly increased in the following 2–3-min time frame (Fig. 2). This occurred because in plants that are irradiated under photorespiratory conditions are shifted to low light or darkness, CO_2_ fixation is immediately attenuated or stops; however, the CO_2_ released in photorespiration continues until all of the metabolic intermediates in the pathway are used^28^.

When the light intensity was switched from low light to high light, the Pn could not recover immediately. It gradually increased and stabilized within 1–2 min (Fig. 2). In *Platanus orientalis* L. leaves, the maximum Pn during the high light period (Pn_max_) was similar to the Pn_S_ and was maintained at a constant level under fluctuating light (Fig. 2, Supplementary Fig. S3). In *Melia azedarach* L. leaves, the Pn_max_ was similar to the Pn_S_ under the low and medium light fluctuation frequencies (the light intensity changed every 4 min or 2 min, respectively), whereas the Pn_max_ decreased gradually under the high light fluctuation frequency (the light intensity changed every 1 min). *Solanum lycopersicum* L. leaves were more sensitive to fluctuating light than the leaves of the other species. The Pn_max_ of *Solanum lycopersicum* L. leaves decreased under fluctuating light, and the decrease was more severe under the high frequency fluctuating light. The minimum Pn during the high light period (Pn_min_) remained constant under fluctuating light for all species (Fig. 2, Supplementary Fig. S3).

To our surprise, the decrease in the Pn_max_ under fluctuating light was only observed for the leaves whose adaxial surface was irradiated. The Pn_max_ of leaves that received abaxial surface irradiation remained constant under fluctuating light (Fig. 2, Supplementary Fig. S3). This result was independent of species and the frequency of the fluctuating light.

Transpiration (E) was similar to Pn for the leaves under fluctuating light. However, E decreased more slowly than Pn during the low light period (Supplementary Fig. S2). Both the maximum and minimum E during the high light period (E_max_, E_min_) remained constant in *Platanus orientalis* L. leaves. These values decreased gradually in *Melia azedarach* L. and *Solanum lycopersicum* L. leaves (Supplementary Figs S2 and S3). The reductions in the E_max_ and E_min_ were aggravated with the increased frequency of fluctuating light. Reductions in the E_max_ and E_min_ were observed under both abaxial and abaxial surface irradiation, but the reductions were much lower under abaxial surface irradiation (Supplementary Figs S2 and S3).

The WUE dramatically decreased during the low light period and increased during the high light period because E changed less and at a slower rate than Pn when the light intensity changed (Supplementary Fig. S2). In contrast to the behaviour of Pn_max_ and E_max_, the WUE_max_ remained constant or increased under fluctuating light (Supplementary Figs S2 and S3). The WUE_min_ also remained constant under fluctuating light for all leaves.

### Integrated gas exchange under simulated adaxial and abaxial surfaces alternant irradiation

**Figure 3:**
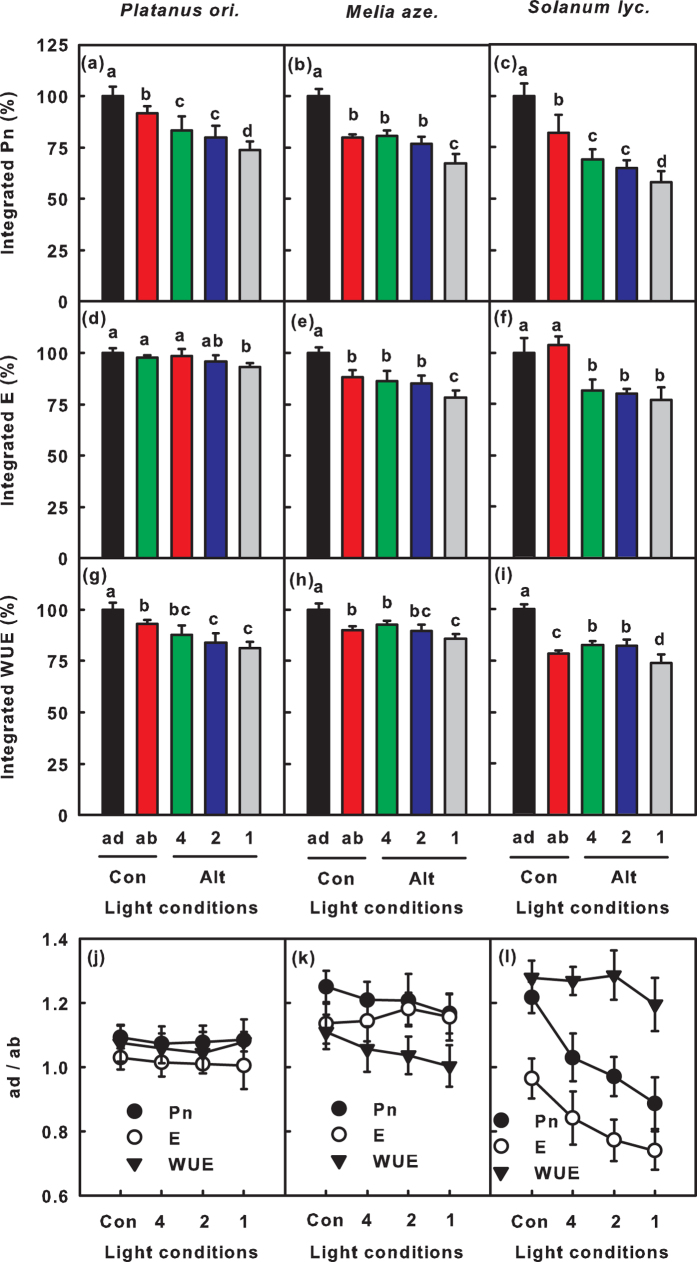
Integrated gas exchange of the whole leaves and the ratio of integrated parameters for the adaxial and abaxial surfaces. (**a–i**) The integrated net photosynthetic rate (Pn, **a**–**c**), transpiration rate (E, **d–f**) and water use efficiency (WUE; **g–i**) of *Platanus orientalis* L. (**a,d,g**), *Melia azedarach* L. (**b,e,h**) and *Solanum lycopersicum* L. (**c,f,i**) under constant irradiation (1,000 μmol m^−2 ^s^−1^) or simulated ad-ab-alt irradiation. (**j–l**) The ratio of the integrated parameter of the adaxial and abaxial surfaces. To simulate alternant irradiation, the adaxial or abaxial surface was irradiated under fluctuating light. The irradiation was switched between high and low light every 4 min, 2 min or 1 min. To calculate the integrated gas exchange under simulated ad-ab-alt irradiation, the values measured during the high light periods of fluctuating light under abaxial and adaxial surface irradiation were summed. The integrated gas exchange under constant irradiation was 100%, whereas the integrated values under simulated ad-ab-alt irradiation were expressed as a percentage of the former value. The reported values are the mean (±SE), n = 5.

Integrated whole leaf gas exchange (the calculation method was showed in *Methods*) under simulated ad-ab-alt irradiation was compared to that resulting from ad-con and ab-con irradiation. The integrated Pn, E and WUE values under simulated ad-ab-alt irradiation were lower than those resulting from ad-con and ab-con irradiation (Fig. 3). This difference increased with an increase in fluctuation frequency. In *Solanum lycopersicum* L. leaves, the integrated Pn and E under high frequency fluctuating light were 42% and 23% lower than those under ad-con irradiation and were 30% and 26% lower than those under ab-con irradiation (Fig. 3).

We also compared the contribution of the adaxial and abaxial surfaces to the gas exchange (ad/ab ratio) under simulated ad-ab-alt irradiation. The ratio between the gas exchange under ad-con and ab-con irradiation served as the control. The ad/ab ratios of gas exchange under simulated ad-ab-alt irradiation and constant irradiation were similar in *Platanus orientalis* L. leaves (Fig. 3). However, the ad/ab ratios of Pn and WUE in *Melia azedarach* L. leaves as well as the ad/ab ratios of Pn and E in *Solanum lycopersicum* L. leaves under simulated ad-ab-alt irradiation were lower than those under constant irradiation and showed a gradual decrease with an increase in fluctuation frequency (Fig. 3).

### Gas exchange induction phase of leaves under high light

**Figure 4:**
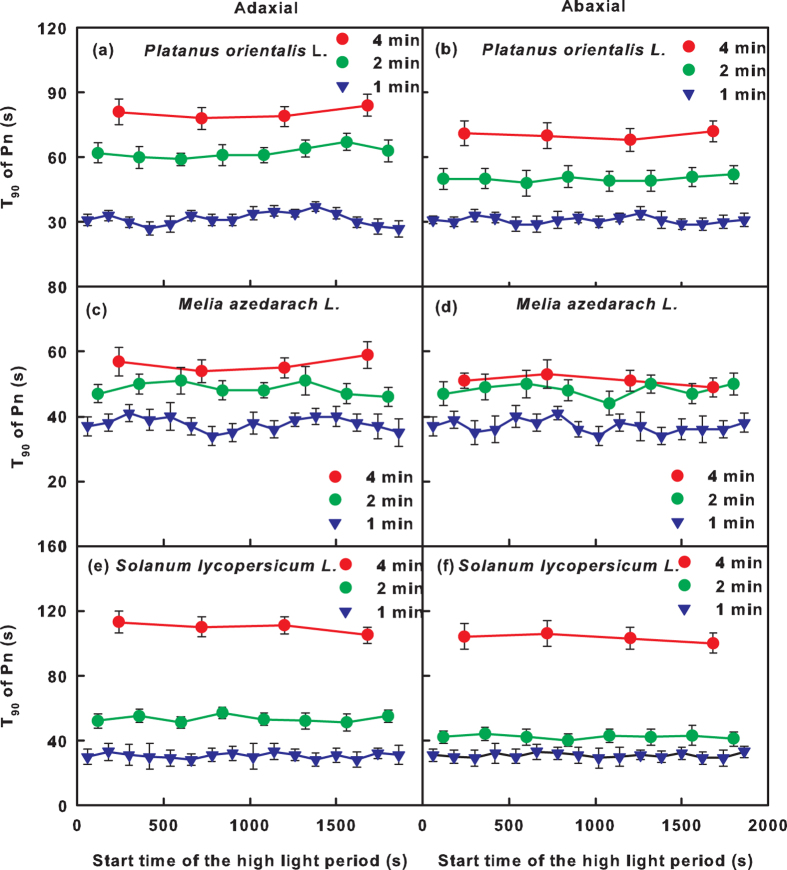
The time required to reach 90% of the maximum net photosynthetic rate (T_90_ of Pn) after the irradiation was changed from low light (100 μmol m^−2 ^s^−1^) to high light (1,000 μmol m^−2^ s^−1^). During the measurement, the adaxial (**a,c,e**) or abaxial surfaces (**b,d,f**) of *Platanus orientalis* L. (**a,b**), *Melia azedarach* L. (**c,d**) and *Solanum lycopersicum* L. (**e,f**) were irradiated by fluctuating light. The irradiation was switched between high and low light every 4 min, 2 min or 1 min. The x-axis indicates the start time of the high light period (as shown in Fig. 1b). Values are the mean (±SE), n = 5.

The time required to reach 90% of the maximum Pn, E and WUE (T_90_) when the light intensity was switched from low light to high light was used to calculate the rates of gas exchange induction. In the leaves from all three species, the T_90_ was constant during simulated ad-ab-alt irradiation but significantly decreased with an increase in fluctuation frequency (Fig. 4, Supplementary Fig. S4). The T_90_ of leaves under abaxial surface irradiation was similar or lower than that of leaves under adaxial surface irradiation (Fig. 4, Supplementary Fig. S4).

### Stomatal limitation and nonstomatal limitation

**Figure 5:**
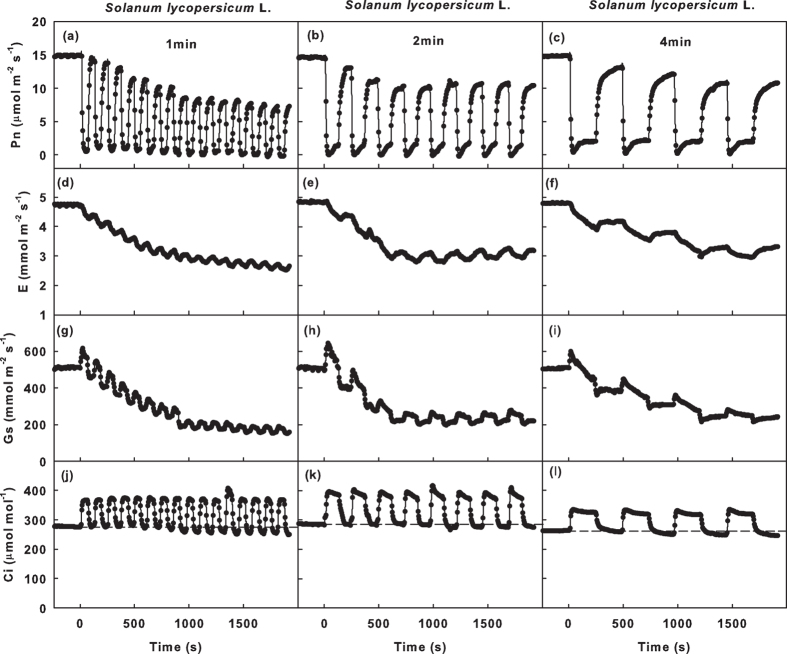
Time course for Pn, E, Gs, Ci in Solanum lycopersicum L. leaves under simulated ad-ab-alt irradiation. Time course for the net photosynthetic rates (Pn; **a–c**), transpiration rate (E; **d**–**f**), stomatal conductance (Gs; **g–i**) and intercelluar CO_2_ pressure (Ci; **j–i**) in *Solanum lycopersicum* L. leaves when the adaxial or abaxial surfaces of leaves were irradiated by fluctuating light, in which the irradiation was switched between high light (1,000 μmol m^−2^ s^−1^) and low light (100 μmol m^−2^ s^−1^) every 1 min (**a,d,g,j**) or 2 min (**b,e,h,k**) or 4 min (**c,f,i,l**). The dotted line shows the Ci under ad-con irradiation.

To clarify the role of stomata in the decrease of Pn_max_ and the delay of Pn induction under simulated ad-ab-alt irradiation, we analysed the Gs, Ci and Pn of *Solanum lycopersicum* L. leaves. When the light intensity switched from low light to high light, the E and Gs increased more slowly than the Pn, and the Ci decreased (Fig. 5). The minimum Ci during the high light period (Ci_min_) decreased with the decrease in Pn_max_ and E_max_ under simulated ad-ab-alt irradiation (Fig. 5).

### Sun leaves and shade leaves

**Figure 6:**
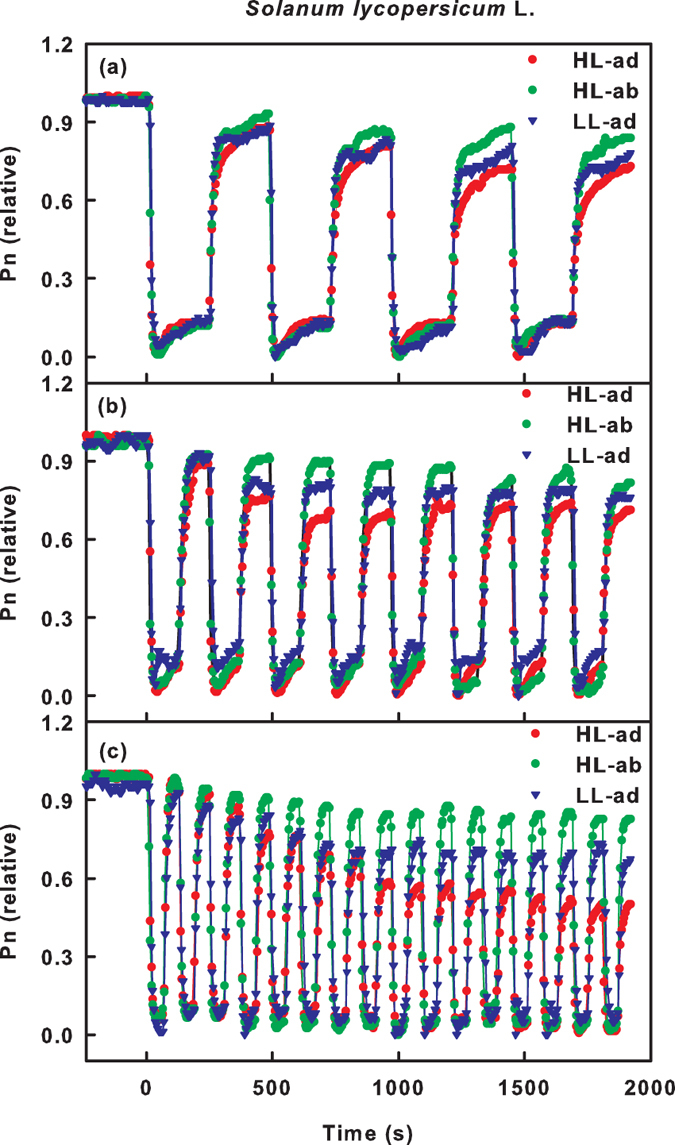
The time course of the net photosynthetic rates (Pn) of *Solanum lycopersicum* L. leaves under high or low light. The adaxial or abaxial surfaces were irradiated by fluctuating light. The irradiation was switched between high light (1,000 μmol m^−2^ s^−1^) and low light (100 μmol m^−2^ s^−1^) every 4 min (**a**), 2 min (**b**) or 1 min (**c**). The initial values of Pn under constant irradiation were considered as 1; other values were calculated as a proportion of the initial values.

The above results demonstrate that the abaxial surface has a lower sensitivity to fluctuating light compared with the adaxial surface. The adaxial surface has characteristics that resemble a classic sun leaf, whereas the abaxial surface has properties that are consistent with shade leaves^3^,^26^,^27^. Therefore, we hypothesized that shade leaf characteristics may contribute to the increased adaptability of the abaxial surface to fluctuating light. To test this hypothesis, we compared the gas exchange of a sun leaf and a shade leaf in response to fluctuating light. The sensitivity of gas exchange to fluctuating light on the adaxial surface of the shade leaf was lower than that of the sun leaf but was still higher than that of the abaxial surface of the sun leaf (Fig. 6). This result demonstrates that the characteristics of a shade leaf contribute to the increased adaptability of the abaxial surface to fluctuating light.

## Discussion

Although some recent reports have investigated dynamic photosynthesis under fluctuating irradiance^22^, the majority of studies were performed on leaves that were irradiated on the adaxial surface^12^,^13^,^14^,^15^,^16^,^17^. Abaxial surface irradiation and ad-ab-alt irradiation, which is ubiquitous in the field, have been neglected^11^. Here, we report the first systematic research focused on the gas exchange of leaves under simulated ad-ab-alt irradiation. The results from this study support the following hypotheses: (1) photosynthetic gas exchange is repressed under simulated ad-ab-alt irradiation and (2) the abaxial surface of the leaf adapts to simulated ad-ab-alt irradiation better than the adaxial surface.

Previous reports regarding dynamic light conditions were focused on sunflecks^21^. Sunflecks provide additional light energy to shaded plants; consequently, sunfleck photosynthesis has always been compared with photosynthesis under shade^21^. However, the total PAR absorbed by the leaves was identical under ad-ab-alt irradiation and ad-con irradiation. The only differences between these two irradiation conditions are the spatial and temporal distributions of light. Therefore, photosynthetic gas exchange under ad-ab-alt irradiation should be compared with that under ad-con irradiation. This study demonstrated that simulated ad-ab-alt irradiation resulted in considerable losses of photosynthetic CO_2_ fixation (Fig. 3).

**Figure 7:**
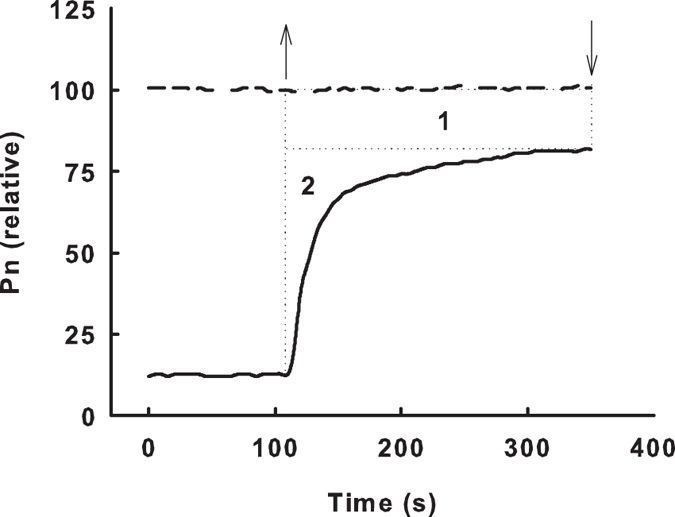
Sketch map of the time course of the net photosynthetic rates (Pn) in response to fluctuating light. The upward arrow shows the leaves were transferred from low light to high light, and the downward arrow shows the leaves were transferred from high light to low light.

The loss of CO_2_ fixation under ad-ab-alt irradiation results from: (1) the reduction in Pn_max_ (region 1 in Figs 7 and 2) the induction requirement of CO_2_ assimilation when the light intensity switches from low light to high light (region 2 in Fig. 7). A previous report suggested that the Pn_max_ under sunflecks could reach a level identical to the steady state Pn under constant irradiation^29^. However, the current study demonstrated that substantial variation exists between species with respect to gas exchange response to a change in light intensity (Figs 2 and 3). In addition, the fluctuating frequency of light intensity critically influences the behaviour of Pn.

The Pn_max_ depends on the balance between the deactivation of the photosynthetic apparatus during low light periods and its reactivation in the following high light periods. It was observed that the T_90_ of Pn remained constant during the 32 min of fluctuating irradiation (Fig. 4). This finding indicated that the rate of photosynthetic activation is steady during high light periods. Consequently, the decreases in Pn_max_ during the simulated ad-ab-alt irradiation occurred because of the deactivation of the photosynthetic apparatus during the low light periods rather than the attenuation of activation during the high light periods.

A reduction in the fluctuation frequency of light intensity results in an extension of both the low light period and the high light period. The extension of the low light period aggravates the deactivation of the photosynthetic apparatus, and the extension of the high light period enhances the activation of the photosynthetic apparatus. The decreases in Pn_max_ were most obvious in leaves exposed to high frequency fluctuating irradiation (Fig. 2, Supplementary Fig. S3). This result suggests that the initial activation rate during the high light period was lower than the initial deactivation rate in the low light periods. However, a reduction in the fluctuation frequency lessened the decrease in Pn_max_ (Fig. 2, Supplementary Fig. S3), which indicates that the balance between the deactivation and activation of the photosynthetic apparatus was related to the duration of the high and low light periods, and the deactivation was more sensitive to the duration compared with the activation.

The photosynthetic activation during the high light periods was reduced with a reduction in the fluctuation frequency, which was reflected by the increase in the T_90_ of Pn (Fig. 4). This result demonstrated that the duration of the low light period not only affects the activation state but also the activation rate of the photosynthetic apparatus in the following high light periods. Previous studies demonstrated that both the deactivation in low light periods and the activation in high light periods consist of two phases. The rapid phase lasts approximately 1 min and is due to the depletion or accumulation of intermediate metabolites^30^. The slow phase, which is in the range of several minutes or more, occurs because light activation of the enzymes in the RuBP regeneration pathway, especially chloroplast fructose-1,6-bisphosphatase (FBPase) and sedoheptulose-1,7-bisphosphatase (SBPase)^21^,^22^, which deactivates more rapid under low light compared with Rubisco^23^,^30^. Under high frequency fluctuating light, the rapid phases of deactivation and activation are dominant. A decrease in the fluctuation frequency results in the occurrence of the slow phase, with a consequent deceleration in photosynthetic activation in the high light periods. Because of the more obvious decrease in Pn_max_ under high frequency fluctuating light (Fig. 2, Supplementary Fig. S3), we suggest that the imbalance between the activation and deactivation was more extreme in the rapid compared with the slow phase.

The gas exchange reached a steady state approximately 20 min after the leaves were exposed to simulated ad-ab-alt irradiation, which was reflected by the steady Pn_max_ (Fig. 2). In this state, the activation and deactivation of the photosynthetic apparatus in the low and high light phases were uniform. It is unclear how this steady state is achieved. It may be due to the adjustment of extra mechanisms or the characteristics of the photosynthetic apparatus. The specific mechanism requires further investigation.

Pn is restricted by mesophyll factors and stomatal factors^25^,^31^. When the light intensity switched from low to high light, Pn and E increased and Ci decreased (Fig. 5). This result suggests that the delay in stomatal opening limited the increase in Pn. In addition, Ci_min_ decreased with a reduction in Pn_max_ and E_max_ (Fig. 5), indicating that the stomatal limitation contributed to the decrease in Pn_max_ under simulated ad-ab-alt irradiation. Thus, the suppression of Pn under simulated ad-ab-alt irradiation was at least partially due to stomatal limitation. In addition, because E was less sensitive than Pn to fluctuating light, the WUE of leaves under simulated ad-ab-alt irradiation was lower than that under constant irradiation (Fig. 2). The decrease in WUE under simulated ad-ab-alt irradiation was entirety due to the delay in induction (Fig. 2, Supplementary Fig. S3); however, the WUE_max_ remained steady or increased slightly (Fig. 2, Supplementary Fig. S3). The above factors demonstrate that the leaves attempted to balance the optimization of Pn and WUE by regulating the stomata under simulated ad-ab-alt irradiation, and the optimization of the WUE was more critical than the maximization of Pn for leaves under simulated ad-ab-alt irradiation. It should be particularly critical for plants in arid and semi-arid areas.

This study also demonstrated that the abaxial surface used fluctuating light more efficiently than the adaxial surface, which was reflected by the fact that the ad/ab ratios of Pn under ad-ab-alt irradiation were higher than the ratio of steady state Pn measured under ad-con and ab-con irradiation (Figs 2 and 3, Supplementary Fig. S3). In particular, when the CO_2_ assimilation of the adaxial surface declined severely under the high frequency fluctuating light, the abaxial surface contributed even more to CO_2_ assimilation than the adaxial surface (Fig. 3l).

Compared with the adaxial surface, the abaxial surface exhibited more rapid photosynthetic induction and maintained a higher Pn_max_ under simulated ad-ab-alt irradiation (Figs 2 and 4). It was also observed that the shade leaves maintained a higher Pn_max_ than the sun leaves under fluctuating light (Fig. 6). Previous studies showed that the rapid induction of photosynthesis during high light periods and the highly activated state of photosynthesis, which is maintained during low light periods, are critical for maximizing light energy under fluctuating light conditions, especially in undergrowth plants^16^,^21^,^22^. Consequently, we suggested that the adaptability of the abaxial surface to fluctuating light occurred because of the shade-leaf-like characteristics of the spongy mesophyll (SM) near the abaxial surface. Indeed, the abaxial surface has characteristics similar to those of shade leaves. It has been reported that plants grown under low-light conditions usually have faster rates of photosynthetic induction, and the loss of the activation state of the photosynthetic apparatus is slower under low light compared with plants grown in high light environments^32^,^33^. Therefore, it is possible that the abaxial surface of the leaf can maintain a higher activation state and more intermediate metabolites under a low level of fluctuating light compared with the adaxial surface.

In the classical view, the SM cells near the abaxial surface contain lower amounts of photosynthetic pigments and proteins^7^,^8^. Therefore, CO_2_ assimilation in SM cells is lower than that in PM cells regardless of which side of the leaf is irradiated^4^,^5^,^6^. However, the shade-leaf-like characteristic enables the abaxial surface to assimilate more CO_2_ under a fluctuating light condition. Consequently, the evolution of a bifacial leaf resulted not only from a resource economy perspective but also because the bifacial leaf could fix more carbon than a leaf with two “sun-leaf-like” surfaces under field conditions.

However, consideration of only the shade-leaf-like characteristic is not sufficient to explain the higher adaptability of the abaxial surface to fluctuating light. This is because the adaptability of the adaxial surface of the shade leaf was lower than that of the abaxial surface of the sun leaf (Fig. 6), even though the adaxial surface of the shade leaf and the abaxial surface of the sun leaf experience similar light environments. In addition, there is substantial variation between species in the adaptability of gas exchange to ad-ab-alt irradiation. *Platanus orientalis* L. is a typical light-demanding upper canopy species. However, contrast to our expectations, both the adaxial and abaxial surfaces of leaves of *Platanus orientalis* L. exhibited perfect adaptability to simulated ad-ab-alt irradiation (Fig. 2). Therefore, the adaptation mechanisms of gas exchange in leaves to ad-ab-alt irradiation remain to be studied further. Moreover, in this study, some factors that may affect the gas exchange of leaves under ad-ab-alt irradiation were neglected, such as the thickness of the leaf and temperature fluctuations. An ad-ab-alt irradiation condition that more closely approximates natural conditions should be considered in future studies.

## Methods

### Plant materials

Two-year-old *Platanus orientalis* L. and *Melia azedarach* L. seedlings and three-month-old *Solanum lycopersicum* L. seedlings were used in this study. The seedlings were grown in 10-L polythene pots containing fertile soil. The seedlings were cultivated in the field, and sufficient water and nutrients were supplied. The maximum light intensity at noon was 1200–1500 μmol m^−2^ s^−1^, and the maximum temperature at noon was 30–33 °C. Half of the *Solanum lycopersicum* L. seedlings were covered with black plastic velamen. The maximum light intensity below the velamen was approximately 100–150 μmol m^−2^ s^−1^. The youngest fully developed leaves were used for the experiments.

### Absorptance

According to a previous description^9^, spectrum (400–700 nm) measurements were performed using a portable Unispec SC spectrometer (PP Systems, USA). Leaf reflectance was measured with a standard bifurcated fibre optic cable, standard leaf clip and the halogen lamp in the spectrometer. To calculate the reflectance, the leaf spectral radiance was divided by the radiance of a 99% reflective white reference standard (Spectralon, Labsphere, North Dutton, NH, USA). Leaf transmittance was measured using two standard straight-fibre optics, a custom-made device and the halogen lamp in the spectrometer. The absorptance was calculated as absorptance = 1 − reflectance − transmittance. The integrated absorptance was the average of the absorptance at different wavelengths between 400 nm and 700 nm.

### Leaf gas exchange measurements

The net photosynthetic rate (Pn), stomatal conductance (Gs), transpiration rate (E), substomatal CO_2_ concentration (Ci) and water use efficiency (WUE = Pn / E) were measured using a CIRAS-3 portable photosynthesis system (PP Systems, Amsbury, MA, USA). Data were automatically recorded by the CIRAS-3 every 5 s. The CO_2_ concentration (380 μmol mol^−1^), relative humidity (60%) and leaf temperature (28 °C) were maintained using an automatic control device on the CIRAS-3. Red-blue light (90%: 10%) was provided by the LED light unit in the CIRAS-3.

The CIRAS-3 cannot irradiate both the adaxial and abaxial surfaces simultaneously. Therefore, we simulated ad-ab-alt irradiation by exposing the abaxial or adaxial surface to fluctuating light separately (Fig. 1b). Before the onset of data collection, the leaves were adapted under high light (1,000 μmol m^−2^ s^−1^) for 20–40 min until Pn and E stabilized. The leaves were then irradiated with constant light or fluctuating light for 32 min. The fluctuating light conditions consisted of a low light (100 μmol m^−2^ s^−1^) and high light (1,000 μmol m^−2^ s^−1^) period that was repeated every 60 s, 120 s or 240 s (Fig. 1b).

The area below the time course curve of the gas exchange represents the integrated gas exchange. The integrated whole leaf gas exchange under simulated ad-ab-alt irradiation was calculated by summing the area for the high light periods under the abaxial and adaxial surface irradiation (the white segments in Fig. 1b).

### Statistical analysis

The absorptance measurements were repeated on 15 different leaves. The gas exchange measurements were repeated using 5 different leaves. The LSD (least significant difference) was used to analyse differences between the different treatments using SPSS 11 (SPSS Inc., Chicago, IL, USA). Different letters indicate significant differences at P < 0.05 (Tukey’s test).

## Additional Information

**How to cite this article**: Zhang, Z.-S. *et al*. Characterization of photosynthetic gas exchange in leaves under simulated adaxial and abaxial surfaces alternant irradiation. *Sci. Rep.* **6**, 26963; doi: 10.1038/srep26963 (2016).

## Supplementary Material

Supplementary Information
