# Supplementary material for: Characterization of photosynthetic gas exchange in leaves under simulated adaxial and abaxial surfaces alternant irradiation
Source: Sci Rep. 2016 Jul 5;6:26963. doi: 10.1038/srep26963 (PMC4932497; doi:10.1038/srep26963)

Title: Characterization of photosynthetic gas exchange in leaves under simulated adaxial and abaxial surfaces alternant irradiation

Zi-Shan Zhang[1,2,3,†], Yu-Ting Li[1,3,†], Hui-Yuan GAO[1,3*], Cheng Yang[4], Qing-Wei Meng[1,2]

[1]State Key Lab of Crop Biology, Tai'an, Shandong Province, China;

[2]College of Horticulture Science and Engineering, Shandong Agricultural University, Tai'an, Shandong Province, China;

[3]College of Life Sciences, Shandong Agricultural University, Tai'an, Shandong Province, China;

[4]Wheat Research Institute, Henan Academy of Agricultural Sciences, Zhengzhou, Henan 450002, China;

[†]These authors contributed equally to this work.

*Corresponding author: Gao Hui-Yuan; Telephone number: 86 538 8245985; E-mail address: gaohy@sdau.edu.cn

Supplementary Figure S1: The steady-state Pn, E, WUE and absorptance of the leaves that received irradiation from the adaxial or abaxial surface. The steady-state net photosynthetic rates (Pn_S; a-c), transpiration rate (E_S; d-f), water use efficiency (WUE_S, g-i) and absorptance to photosynthetic active radiation (400-700nm; abs; j-l) in leaves of *Platanus orientalis* L. (a, d, g, j), *Melia azedarach* L. (b, e, h, k) and *Solanum lycopersicum* L. (c, f, i, l) when either adaxial (ad) or abaxial (ab) surfaces of leaves were irradiated by 1,000 μmol m^{-2} s^{-1} light. Different letters indicate significant differences between adaxial and abaxial surfaces irradiation. Values are means (± SE), n = 5 (for Pn_S, E_S and WUE_S) or 15 (for abs).

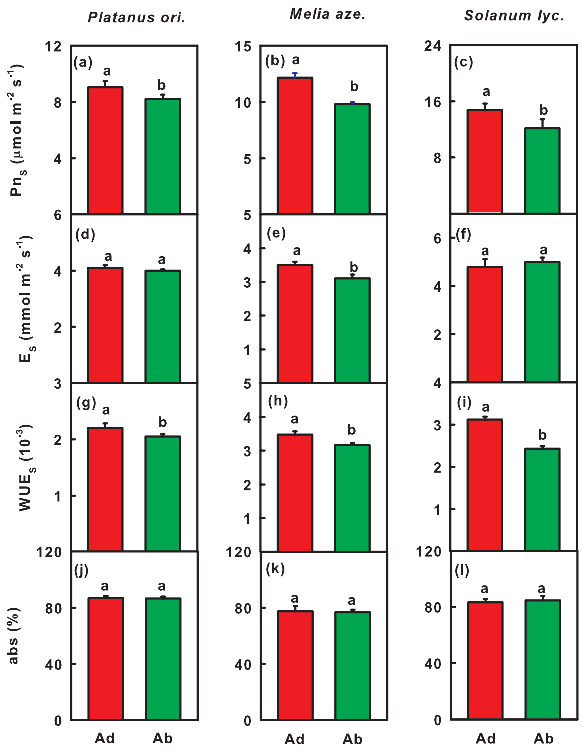

Supplementary Figure S2: Time course of the transpiration rate and water use efficiency of leaves under simulated ad-ab-alt irradiation. Time course for the transpiration rate (E) and water use efficiency (WUE) in leaves of *Platanus orientalis* L., *Melia azedarach* L. and *Solanum lycopersicum* L. when the adaxial or abaxial surfaces of leaves were irradiated by fluctuating light, in which the irradiation was switched between high light (1,000 μmol m^{-2} s^{-1}) and low light (100 μmol m^{-2} s^{-1}) every 4 min or 2 min or 1 min.

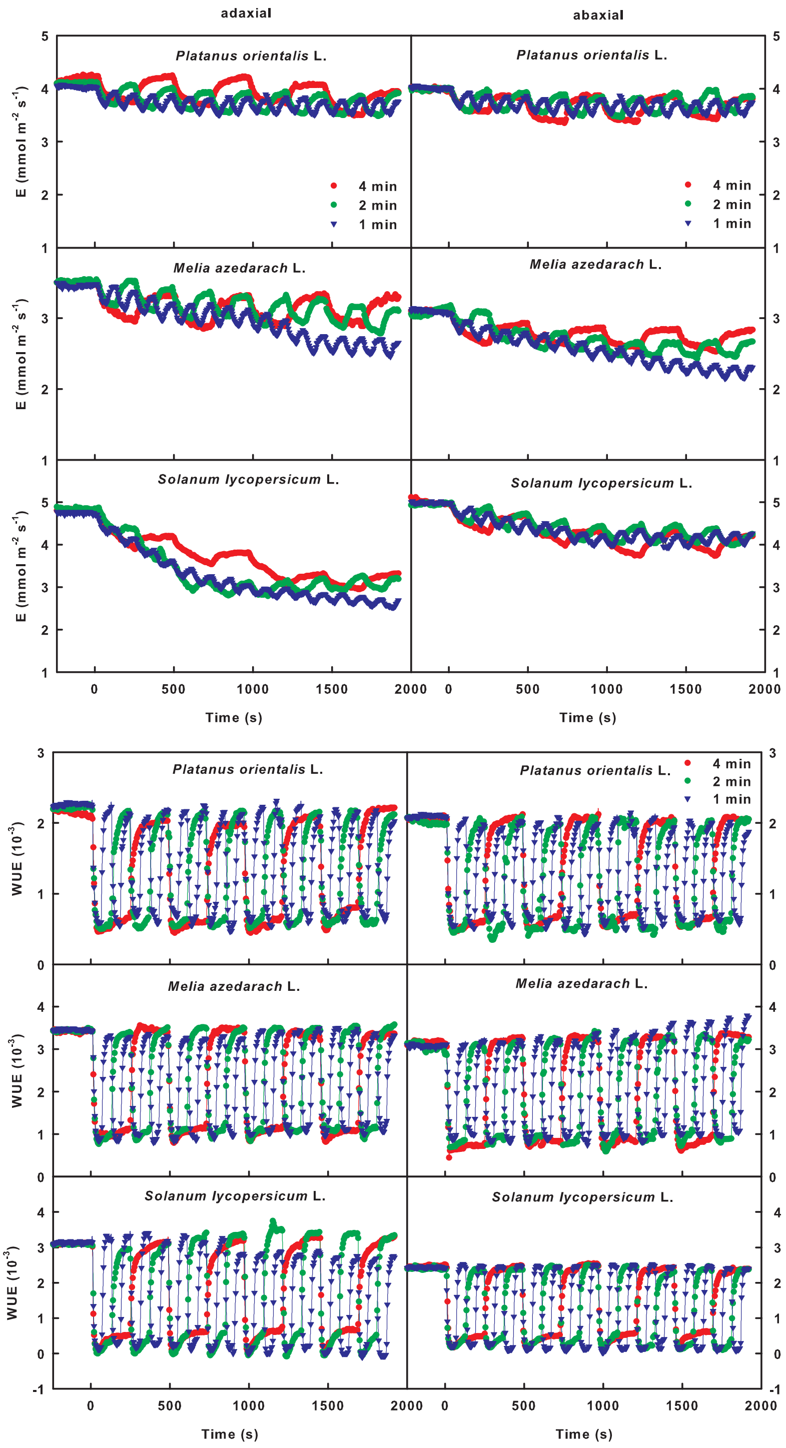

Supplementary Figure S3: The maximum Pn, E and WUE during the high light period and the minimum E during the low light period under simulated ad-ab-alt irradiation. The maximum of net photosynthetic rate (Pn_{max}), transpiration rate (E_{max}) and water use efficiency (WUE_{max}) during the high light period of fluctuating irradiation, and the minimum of E (E_{min}) during the low light period of fluctuating irradiation. During measurement, the adaxial and abaxial surfaces of leaves from *Platanus orientalis* L., *Melia azedarach* L. and *Solanum lycopersicum* L. were irradiated by fluctuating light, in which the irradiation was switched between low light (100 μmol m^{-2} s^{-1}) and high light (1,000 μmol m^{-2} s^{-1}) every 4 min or 2 min or 1 min. Values are means (± SE), n = 5.

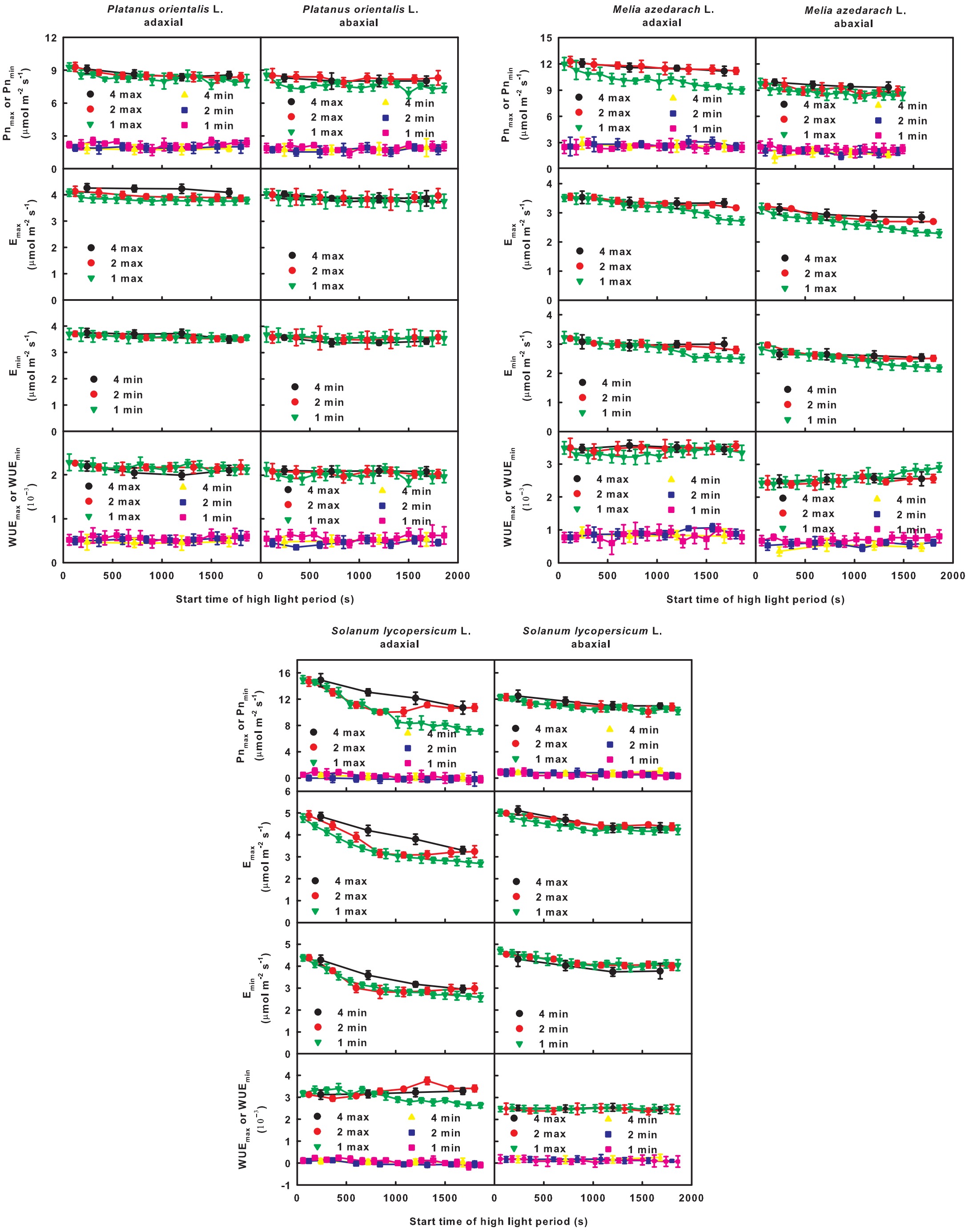

Supplementary Figure S4: The time required to reach 90% of the maximum transpiration rate (T_{90} of E) and water use efficiency (T_{90} of WUE) after the irradiation was shifted from low light to high light. During measurement, the adaxial and abaxial surfaces of leaves from *Platanus orientalis* L., *Melia azedarach* L. and *Solanum lycopersicum* L. were irradiated by fluctuating light, in which the irradiation was switched between high (100 μmol m^{-2} s^{-1}) and low light (100 μmol m^{-2} s^{-1}) every 4 min or 2 min or 1 min. Values are means (± SE), n = 5.

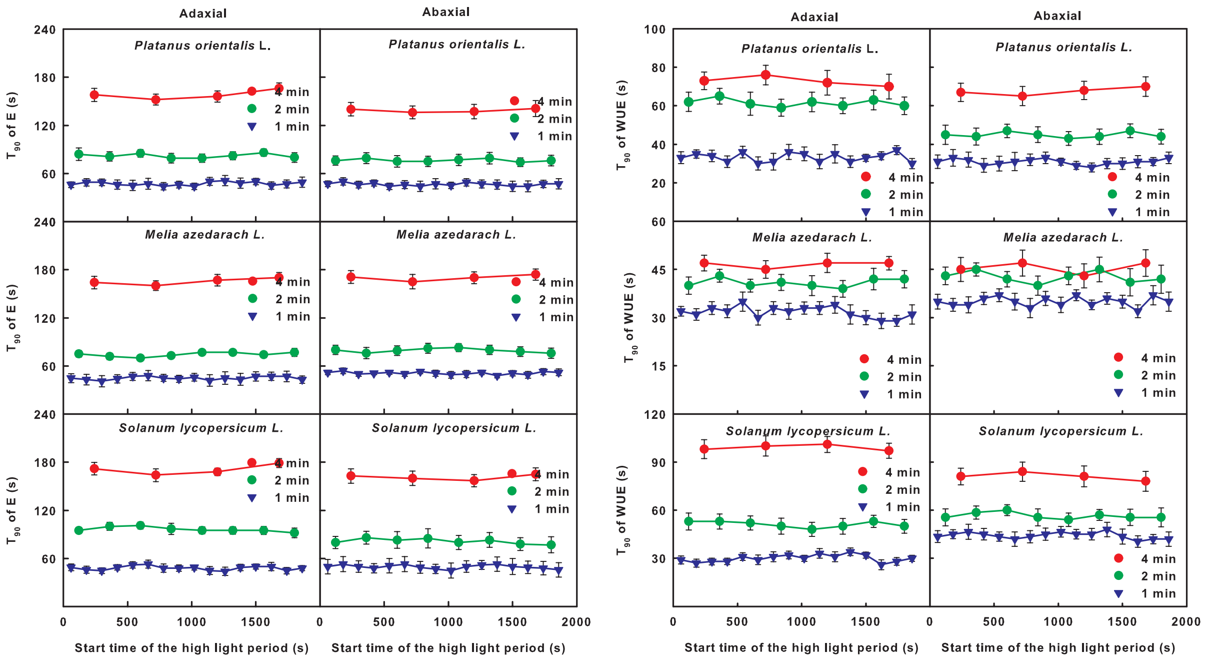

Supplement: Supplementary Information [file srep26963-s1.pdf]
